# Individualized supervised resistance training during nebulization in adults with cystic fibrosis

**DOI:** 10.12669/pjms.325.9960

**Published:** 2016

**Authors:** Ina Shaw, Janine E. Kinsey, Roxanne Richards, Brandon S. Shaw

**Affiliations:** 1 Prof. Ina Shaw, PhD. Department of Sport & Movement Studies, University of Johannesburg, Republic of South Africa; 2 Ms. Janine E. Kinsey, B.Hons. Department of Sport & Movement Studies, University of Johannesburg, Republic of South Africa; 3 Mrs. Roxanne Richards, B.Hons. Department of Sport & Movement Studies, University of Johannesburg, Republic of South Africa; 4 Prof. Brandon S. Shaw, PhD. Department of Sport & Movement Studies, University of Johannesburg, Republic of South Africa

**Keywords:** Cardiopulmonary Disease, Health-Related Quality of Life, Lung Disease, Strength Training, Weight Training

## Abstract

**Background & Objective:**

Since dyspnea limits exercise adherence and intensity in cystic fibrosis (CF) patients, engaging in resistance training (RT), which causes less dyspnea than other exercise modalities, while using nebulizers could not only overcome this barrier, but also enhance long-term adaptations to treatment. The objective of this study was to examine the effects of RT during nebulization on spirometry, anthropometry, chest wall excursion, respiratory muscle strength and health-related quality of life (HRQOL).

**Methods:**

Fourteen male and female CF patients were assigned to a four-week, 20-minute, 5-day per week proof-of-concept RT group (RTG) (n=7) or non-exercising control group (CON) (n=7), with 3 CON patients later dropping out of the study. Patients performed whole body exercises for 3 sets of 10 reps using resistance bands, since such bands have previously demonstrated a greater effect on functional exercise capacity than conventional RT in lung patients.

**Results:**

The RTG displayed significant (p≤0.05) increases in FEV_1_, FEV_1_/FVC, latissimusdorsi strength, pectoralis major clavicular portion strength, pectoralis major sternocostal portion strength and emotional and digestion HRQOL domains, while decreasing pectoralis minor strength on the left and social, body image and respiration HRQOL domains.

**Conclusion:**

This small scale proof-of-concept investigation demonstrates the multiple and simultaneous benefits of RT during nebulization in CF patients. The improvements in pulmonary measures are particularly promising especially since this study only made use of a four-week experimental period. This study provides an important alternative, time-saving treatment for the CF patient that does not add to the treatment burden of CF patients.

## INTRODUCTION

Cystic fibrosis (CF) is a multi system disease that primarily affects the pulmonary system and pancreas. Pulmonary abnormalities arising from CF include excess production of viscous mucus secretions, which may obstruct airways, leading to subsequent declines in pulmonary function.^1^ While CF has no cure, the goal of treatments for CF include the preventing and controlling of lung infections, the loosening and removing thick, sticky mucus from the lungs and improvements in physical functioning and quality of life (QOL).

However, from infancy, CF patients must be subjected to all kinds of stressful and/or time-consuming treatments, such as inhalation therapy, mist tents, suction machines, physical rehabilitation and nebulization.^2^ While treatments have greatly improved in recent years, standards of care for patients with CF have been defined largely on the basis of best practice, and are given to treatment regimens showing low chronic pulmonary infection rates, greatest patient longevity, and least patient morbidity.^3^ However, it is becoming more critical that treatments be scientifically proven and since CF patients have multiple treatments in a day, timesaving in the development of treatments is crucial.

Taking the primary goal of CF treatment into account, nebulizers are commonly used for the treatment of CF and other respiratory diseases and use oxygen, compressed air or ultrasonic power to break up medical solutions and suspensions into small aerosol droplets that can be directly inhaled. The therapeutic effectiveness of an inhaled drug depends on a number of factors, but in particular how much bypasses the oropharynx and deposits in the lungs.^4^ While inertial impaction occurs in the upper airways and in the first few generations of bronchi, distally, as the cross-sectional area of the airways increases, airflow velocity decreases and becomes more laminar and deposition of the inhaled submicronic particles becomes dependant on gravitational sedimentation^4^, provided that they are not exhaled.^5^ As such, it may prove important for CF patients to engage in techniques that slow and deepen tidal breath, thus increasing dwell time.

One such technique that may prove effective is resistance training (RT). This is because RT as an exercise modality makes use of Valsalva manoeuvres which not only increases the depth, but also slows the rate of tidal volume and thus limits the chances of expelling the inhaled submicronic particles. The concurrent use of RT would not only increase the efficacy of nebulization, but may itself provide additional benefits to the CF patient without resulting in an additional time burden on CF patients. In turn, the use of nebulization during RT could reduce exercise-induced hypoxemia and increase exercise tolerance, oxygen supply and adaptations^6^ and thus overcome the primary reason that many CF patients abstain from exercise. Interestingly, since dyspnoea limits the exercise adherence and intensity, RT, which causes less dyspnoea^7^, it could be an alternative mode of exercise for CF patients. The aim of this study was to examine the long-term effects of RT during nebulization on spirometry, anthropometry, chest wall excursion, respiratory muscle strength and health-related quality of life (HRQOL).

## METHODS

A convenience sample of 14 CF male and female patients, aged between 16 and 33 years, attending the Cystic Fibrosis Clinic at the Charlotte Maxeke Johannesburg Academic Hospital in Johannesburg, South Africa that were approved by their attending specialists for participation in the study. They were not engaging in any regular exercise program were assigned to either a resistance training group (RTG) (n=7) or non-exercising control group (CON) (n=7) for this small scale proof-of-concept investigation. Prior to participation, all patients gave written, informed consent to participate in the study.

The study was approved by the Research Ethics Committee of the University of Johannesburg (AEC01-69-2014), the University of Witwatersrand Human Research Ethics Committee (Medical) (M140628) and the director of Clinical Services Charlotte Maxeke Johannesburg Academic Hospital. Patients were required to be free from any acute respiratory infection symptoms or pulmonary exacerbations, current or previous participation within the last six months in exercise therapy or changes in any therapy four weeks prior to the evaluations. Patients were excluded from participation in the study if they presented with relative and absolute contraindications to exercise or presented with any clinically significant abnormalities that could have interfered with the assessments. All patients underwent identical pre- and post-test measurements.

Since the life expectancy of CF patients in developing countries is much less than their counterparts in developed countries, the present study made use of simple measuring procedures. This is because since CF is not a priority for governments in developing countries, there are not only limited resources available, but a lack of trained health professionals for the care of specific problems in CF patients.^8^

The present study made use of the Cystic Fibrosis Questionnaire-Revised (CFQ-R) which is a disease-specific health-related qualify of life (HRQOL) measure assessing (1) physical, (2) emotional, (3) social, (4) body image, (5) eating, (6) treatment burden, (7) respiration and (8) digestion domains.^9^

Body mass and stature were measured to the nearest 0.1 kg and 0.01 meters (m), respectively using a scale and standard wall-mounted stadiometer (Mettler DT Digitol, Mettler-Toledo AG, Ch-8606 GreiFensee, Switzerland) with patients wearing minimal clothing and no shoes. Skinfolds of the triceps, subscapular, suprailiac, paraumbilicus, frontal thigh and medial calf were measured to the closest 0.2 millimeters (mm) using a Harpenden skinfold caliper (Rosscraft Industries, Canada). Percentage body fat was calculated using the equations of Withers et al.^10^, fat mass by multiplying body mass with percentage body fat which was divided by 100, lean mass as total body mass in kg subtracted by fat mass in kg and body mass index (BMI) by dividing body mass by stature squared (body mass/stature^2^) and expressed as kilograms per square meter (kg.m^-2^).^11^

Spirometry of all patients were performed with patients upright and wearing a nose clip so that no air could escape during the test. A mouthpiece was inserted into the turbine sensor (Quark PFTergo, Cosmed, Rome, Italy), where the patients were blowing into. The FVC test required each patient to expire as hard and as fast as possible and had to complete the cycle by inspiring as hard and rapidly as possible. The tests were performed at least three times with the largest value recorded being utilized in the final analysis.^12^

Since it is essential for CF patients to maintain and improve mobility of the chest wall, abdominal and thoracic dimensions and kinematics measurements were used as an evaluative method for diaphragmatic breathing excursion to quantify possible alterations in thoracic capacity and abdominal and chest wall compliance as achieved by all expiratory and inspiratory muscles.^13^ By measuring the abdomen and thorax with a nondistendable measuring tape in centimetres to the nearest 0.1 cm during rest, maximal inspiration and expiration over the second intercostal space, xiphoid process and mid-point between the xiphoid process and umbilicus, a competency in diaphragmatic breathing can be demonstrated by a reduction in rib cage excursion. This evaluation was used to quantify possible alterations in thoracic capacity and possibly abdominal and chest wall compliance as achieved by all inspiratory and expiratory muscles.^13^

The present study assessed the respiratory muscles for maximal effort due to the major limitation of spirometry in its poor sensitivity to detect moderate inspiratory muscle weakness^14^, the strength of the accessory respiratory muscles, and specifically the latissimus dorsi, pectoralis major (sternocostal and clavicular portions), pectoralis minor and abdominals, were manually tested using an aneroid sphygmomanometer (Alpk2 Sphygmomanometer, Japan) for the lattisimus dorsi, pectoralis major (clavicular portion), pectoralis major (sternocostal portion), pectoralis minor muscles, held by the evaluator at the test position and the difference achieved by the patients from a baseline setting of 20 millimetres mercury (mmHg) and the seven stage sit-up test for abdominal muscles.^13^,^15^,^16^

As CF patients display reduced exercise tolerance and complaints of fatigue and dyspnea, even after minimal exertion, the present study utilised a supervised 20-minute, 5 days per week (Monday through Friday) progressive RT program for four weeks^18^ with elasticated resistance bands (Thera-Band®) targeting large muscle groups, since such bands have previously demonstrated a greater effect on functional exercise capacity than conventional resistance training in COPD patients.^18^ Patients performed 3 sets of 10 reps for (1) seated chest press, (2) squat, (3) lateral raises, (4) bicep curls, (5) plank, (6) standing high rows, (7) shrugs, (8) latissimusdorsi pull downs and (9) rhomboid squeezes with rest periods of 30 seconds or less.^19^ While different band tensions were utilised for different exercises with greater resistance required for larger muscle groups, regardless of the colour (resistance) of the band, all strength training exercises were performed with the bands elongated to at least 70%.^20^ This protocol was designed to ensure that all patients trained with a consistent and progressive amount of resistive force. In an attempt to reduce exercise-induced hypoxemia and increase exercise tolerance, oxygen supply and adaptations, the exercise program was during the patients’ nebulizing times.^6^ In addition, this RT and nebulization protocol was performed concurrently in an attempt to relieve the time-burden of treatments on CF patients. The CON patients were instructed to maintain their normal daily activities and medication usage throughout the four-week experimental period and received no prescribed exercise program.

Data were analysed using the Statistical Package of Social Sciences (SPSS) Version 20 (IBM Corporation, Armonk, NY). Statistical analysis consisted of basic statistics to determine baseline and post-training means and standard deviations. T-tests were utilised to determine if a significant change occurred within groups from pre- to post-test and between groups. A probability value of ≤ 0.05 was considered significant.

## RESULTS

While 14 male and female CF patients were assigned to the four-week treatment protocol, 3 CON patients were not included in the final study due to their failure to attend the post-test evaluations.

Results indicated significantly (p≤0.05) increased FEV_1_ (p=0.021), FEV_1_/FVC (p=0.046), latissimusdorsi strength on the left (p=0.022) and right (p=0.001), pectoralis major: clavicular portion strength on the left (p=0.042) and right (p=0.038), pectoralis major: sternocostal portion strength on the left (p=0.030) and right (p=0.025) and emotional (p=0.011) and digestion (p=0.018) HRQOL domains on the CFQ-R in the RTG (Table-I). A significantly decreased pectoralis minor strength on the left (p=0.048) and social (p=0.032), body image (p=0.046) and respiration (p=0.028) HRQOL domains on the CFQ-R were also found in the RTG.

**Table-I:** Effect of resistance training on pulmonary function, anthropometry & quality of life in patients with cystic fibrosis.

	*Resistance Training Group (RTG) (n=7)*		*Non-Exercising Control Group (CON) (n=4)*	

	*Pre-test*	*Post-test*	*Pre-test*	*Post-test*
ANTHROPOMETRY				
Body mass (kg)	53.76±13.19	53.90±13.12	64.25±30.71	64.50±29.13
% Body fat (%)	15.51±5.78	16.51±5.98	18.12±11.38	18.05±10.82
Lean mass (kg)	49.60±7.71	44.99±9.80	50.28±19.31	50.65±17.49
Fat mass (kg)	8.61±4.65	8.87±4.77	13.95±12.49	14.10±11.86
SPIROMETRY				
FEV_1_ (ℓ.sec^-1^)	1.53±0.72	1.96±1.15*	2.43±0.92	2.50±1.05†
FVC (ℓ.sec^-1^)	2.53±0.86	2.78±1.20	3.70±1.47	3.62±1.50
FEV_1_/FVC	58.27±13.00	63.26±15.61*	67.20±10.40	68.70±5.64†
ABDOMINAL AND THORACIC KINEMATICS				
Resting chest (cm)	83.60±11.59	82.57±10.47	88.50±16.92	85.88±18.92
Chest inspiration (cm)	87.00±12.86	85.57±9.62	88.10±10.47	89.23±18.69
Chest expiration (cm)	82.33±18.58	81.06±10.09	90.67±16.56	83.73±17.42
MUSCULAR STRENGTH				
Latissimusdorsi (L) (mmHg)	50.57±16.44	77.71±30.32*	57.00±27.25	81.50±54.22†
Latissimusdorsi (R) (mmHg)	44.00±23.21	80.29±25.68*	41.50±24.08	78.00±48.19†
Pectoralis major: clavicular (L) (mmHg)	60.00±34.16	75.14±21.60*	52.50±36.01	96.75±53.25†
Pectoralis major: clavicular (R) (mmHg)	59.14±31.81	75.14±23.60*	58.00±32.08	109.50±67.54†
Pectoralis major : sternocostal (R) (mmHg)	82.57±34.25	102.57±25.16*	74.50±48.51	80.50±42.34†
Pectoralis major: sternocostal (R) (mmHg)	89.43±40.16	109.14±28.47*	76.50±40.61	102.50±68.50†
Pectoralis minor (L) (mmHg)	75.14±56.35	64.29±24.64*	41.00±15.19	66.50±36.78*†
Pectoralis minor (R) (mmHg)	74.29±49.95	69.14±20.03	45.00±21.57	63.50±25.42*†
Seven-stage abdominal test	1.29±1.38	1.57±1.13	3.00±2.45	2.01±0.82*†
QUALITY OF LIFE				
Physical (#)	38.88±28.86	42.06±21.96	65.28±44.99	44.44±22.68*
Emotional (#)	39.28±26.99	48.81±5.22*	66.66±20.69	43.76±5.39*†
Social (#)	57.14±27.22	44.21±15.96*	69.05±29.74	64.28±21.47
Body image (#)	65.07±26.00	46.03±23.51*	66.66±24.00	41.66±10.64*
Eating (#)	49.20±12.60	46.03±25.20	63.88±21.03	25.00±29.22*
Treatment Burden (#)	60.31±29.29	55.55±18.14	58.33±33.18	52.77±13.98
Respiration (#)	52.38±26.66	33.33±15.21*	64.58±17.18	35.42±26.68*
Digestion (#)	52.38±32.53	71.43±29.99*	75.00±16.67	91.67±16.67*

Values are means±standard deviation;

* p≤0.05 compared to pre-test;

† p ≤ 0.05 resistance training group (RTG) compared to non-exercising control group (CON) kg: kilograms; %: percent; BMI:FEV_1_: Forced expiratory volume in one second; FVC: Forced vital capacity;ℓ.sec-1: litres per second; cm: centimeters; R: right; L: left; mmHg: millimeters mercury;

# is the score out of 100 (100 being the highest score possible and 0 being the lowest).

In the CON, pectoralis minor strength on the left (p=0.034) and right (p=0.042), and the digestion (p=0.026) HRQOL domain on the CFQ-R increased significantly following the experimental period. In addition, the CON displayed significant decreases in physical (p=0.029), emotional (p=0.030), body image (p=0.036), eating (p=0.008) and respiration (p=0.018) HRQOL domains on the CFQ-R.

Results also indicated that FEV_1_ (p=0.041), FEV_1_/FVC (p=0.032), latissimusdorsi strength on the left (p=0.047), pectoralis major: sternocostal portion on the right (p=0.025), pectoralis minor strength on the left (p=0.011) and right (p=0.020), seven-stage abdominal test (p=0.001) and emotional (p<0.001) HRQOL domain on the CFQ-R were significantly different at post-test when comparing the RTG and CON.

## DISCUSSION

From infancy, CF patients are subjected to numerous stressful and/or time-consuming treatments, such as inhalation therapy, mist tents, suction machines, exercise-based rehabilitation and nebulization.^2^ Specifically relating to exercise-based rehabilitation, aerobic exercise training is a commonly used approach for improving physical capacity and HRQOL in pulmonary rehabilitation programs.^21^ However, since dyspnea limits exercise adherence and intensity in lung patients, the use of RT may be warranted in this population. This is because RT results in less dyspnea than other forms of exercise. In addition, the concomitant use RT during nebulization times may result in improvements in both the efficacy of the nebulization, since RT may increase the dwell time of the inhaled submicronic particles as a result of Valsalva maneuvers, and the enhanced efficacy of the nebulized medications may reduce exercise-induced hypoxemia and increase exercise tolerance, oxygen supply and adaptations and thus overcome the primary reason that many CF patients abstain from exercise and enhance long-term adaptations.

In this regard, this study has demonstrated that RT during nebulization times provides simultaneous benefits in spirometry, muscle strength and QOL. The improvements in spirometry in this study, especially considering the short duration of the exercise program, are noteworthy since increases in spirometry in lung patients following exercise training are generally uncommon^19^, even in CF patients.^22^ However, since FEV_1_ is considered an effort-/force-/muscle-dependent variable, any improvements in respiratory muscle strength, as also found in this study, are also likely to result in improvements in lung function.^23^ Although few data exist on the effect of exercise on respiratory muscle function in patients with CF,^24^ the present study demonstrated that a RT program can improve the strength of the accessory muscles of respiration needed when an individual breathes in at the maximal flow rate.

Following a period of RT, the present study found increases in the emotional and digestion domains on the CFQ-R. This is a novel finding in that quality of life has only previously been correlated with an individual’s aerobic capacity.^25^ However, it must be noted that social, body image and respiration domains of HRQOL decreased following the RT. Despite an improvement in the emotional domain, the decrease in the social domain is not entirely unanticipated in that depressive symptoms are common in individuals with CF^25^ and further investigation is warranted on the effect of RT on mood state and psychological domains of HRQOL. While the finding on the respiration domain is not congruent with the findings regarding spirometry, the finding on the body image domain is not unexpected considering that the experimental group did not demonstrate any improvements in body composition following the exercise training. This is a unique finding in that previously, research has demonstrated that RT does not improve HRQOL in children with CF.^1^

However, caution must be heeded that the results of this study cannot be generalized to all patients with CF since the convenience sample of 14 is too small (even though it made use of a control group) to reach a definitive conclusion and the CON and RTG baseline values, especially spirometry and HRQOL, may be negatively correlated with change since the RTG patients with low scores at baseline would typically improve more than those with high scores. In addition, the study found simultaneous improvements and decreases in some measures warranting the need for further research. These future studies should utilize longer durations of exercise-based rehabilitation especially when evaluating HRQOL.

## CONCLUSION

These results of this small scale proof-of-concept investigation suggest that CF patients should undertake regular RT during nebulization to ensure higher indices of pulmonary function, respiratory muscle strength and some aspects of HRQOL. This study also highlights the importance of respiratory muscular strength development in this population, considering that improvements in spirometry are generally uncommon in CF patients following exercise training. This study potentially provides an effective alternative, time-saving treatment for the CF patient that does not add to the treatment burden.
